# A Rare Metastasis to the Stomach From Cervical Cancer: A Case Report and Review of the Literature

**DOI:** 10.7759/cureus.94889

**Published:** 2025-10-18

**Authors:** Srived Meda, Balaji Musunuri, Shiran Shetty, Karthik Udupa, Deepak Nayak

**Affiliations:** 1 Department of Gastroenterology and Hepatology, Kasturba Medical College, Manipal, Manipal Academy of Higher Education, Manipal, IND; 2 Department of Medical Oncology, Kasturba Medical College, Manipal, Manipal Academy of Higher Education, Manipal, IND; 3 Department of Pathology, Kasturba Medical College, Manipal, Manipal Academy of Higher Education, Manipal, IND

**Keywords:** cervical cancer, gastric metastasis, gastric outlet obstruction, gastric tumors, gi bleed, nonkeratinising, squamous cell carcinoma, stomach secondaries

## Abstract

Cervical cancer is one of the leading causes of cancer-related mortality among females. The common sites of metastasis from cervical cancer include the lungs, bones, and liver. Gastric metastases are rare and are associated with poor prognosis. Gastric metastases most commonly arise from breast and lung cancers. However, metastasis from primary cervical cancer is extremely rare and has been reported only in a few cases in the literature to date. We present the case of a 55-year-old female with a history of advanced cervical cancer who presented with features of gastric outlet obstruction and gastrointestinal bleeding. Endoscopy revealed an infiltrating mass in the stomach and pylorus/duodenum, with biopsy confirming squamous gastric carcinoma of metastatic origin. When patients with cervical cancer present with upper gastrointestinal symptoms, the possibility of metastatic gastric involvement should be considered.

## Introduction

The stomach is an uncommon site of gastrointestinal tract metastasis, with literature limited to case reports or series and a reported incidence of 0.2-0.7% based on clinical and autopsy findings. The most common primary tumors that spread to the stomach are breast and lung cancers, while cervical cancer metastasis to the stomach is exceedingly rare [1]. These cases have a poor prognosis and may present with symptoms such as loss of appetite, abdominal pain, fatigue, nausea, and vomiting.

Cervical cancer is one of the most common malignancies of the female genital tract and is the second leading cause of cancer mortality in women aged 20 to 39 years [2]. While early-stage or locally advanced cervical cancer has a better prognosis compared to advanced stages, where the median survival is 8-13 months, distant metastasis can occur. Common sites of distant metastases of cervical cancer include the lungs, bone, and liver [2]. In contrast to local invasion, metastatic cervical cancer presents with nonspecific complaints. We present a rare case that highlights an unusual cause of cervical cancer metastasizing to the stomach, resulting in gastric outlet obstruction and gastrointestinal bleeding. In this report, we describe a patient with cervical cancer who presented with signs and symptoms of gastric outlet obstruction and upper gastrointestinal bleeding. Further evaluation revealed gastric metastasis, with biopsies confirming metastatic squamous cell carcinoma. We also reviewed the literature to date to discuss reported cases of gastric metastasis arising from cervical cancer.

## Case presentation

A 55-year-old female presented to the gastroenterology department with intermittent abdominal pain, predominantly in the left hypochondrium, associated with vomiting for two months and six to seven episodes of hematemesis (coffee-ground vomitus). She was a known case of metastatic cervical cancer diagnosed two years prior. She had completed six cycles each of first- and second-line chemotherapy regimens: bevacizumab/carboplatin/paclitaxel and topotecan/cisplatin, respectively. On examination, her vitals were stable, and mild abdominal tenderness was noted in the epigastric region on palpation. Routine laboratory investigations showed no abnormalities except for anemia (hemoglobin 9.2 g/dL). She was suspected of having an upper GI bleed and possible ulcer disease. In view of her symptoms, she was taken up for upper GI endoscopy.

**Figure 1:**
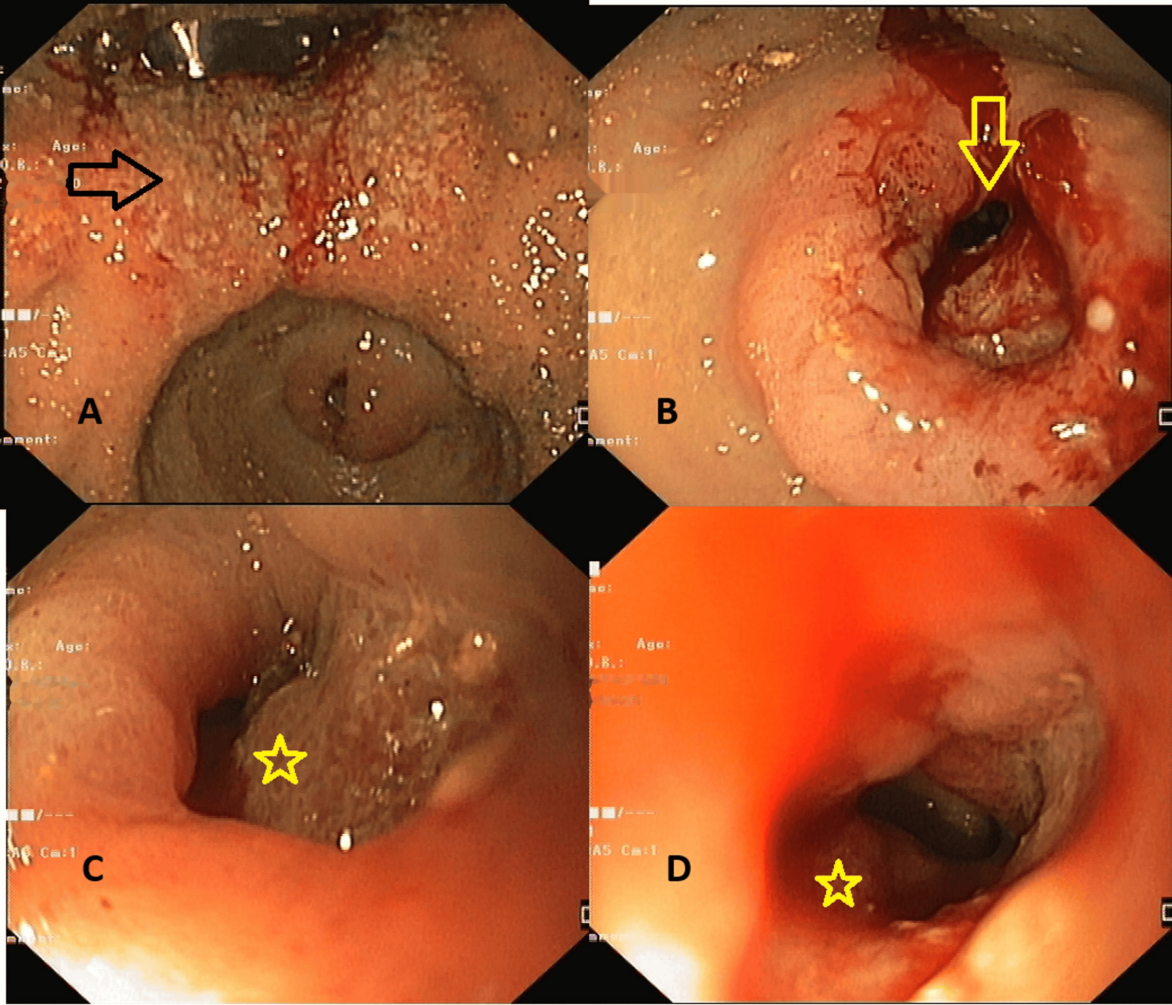
Endoscopic images showing a malignant lesion in the stomach and pylorus/duodenum. Endoscopic images showing an irregular friable ulcerated malignant lesion (black arrow) in stomach (A), another friable lesion at pylorus/duodenum (yellow arrow in B) causing stricture (yellow star in C and D).

**Figure 2:**
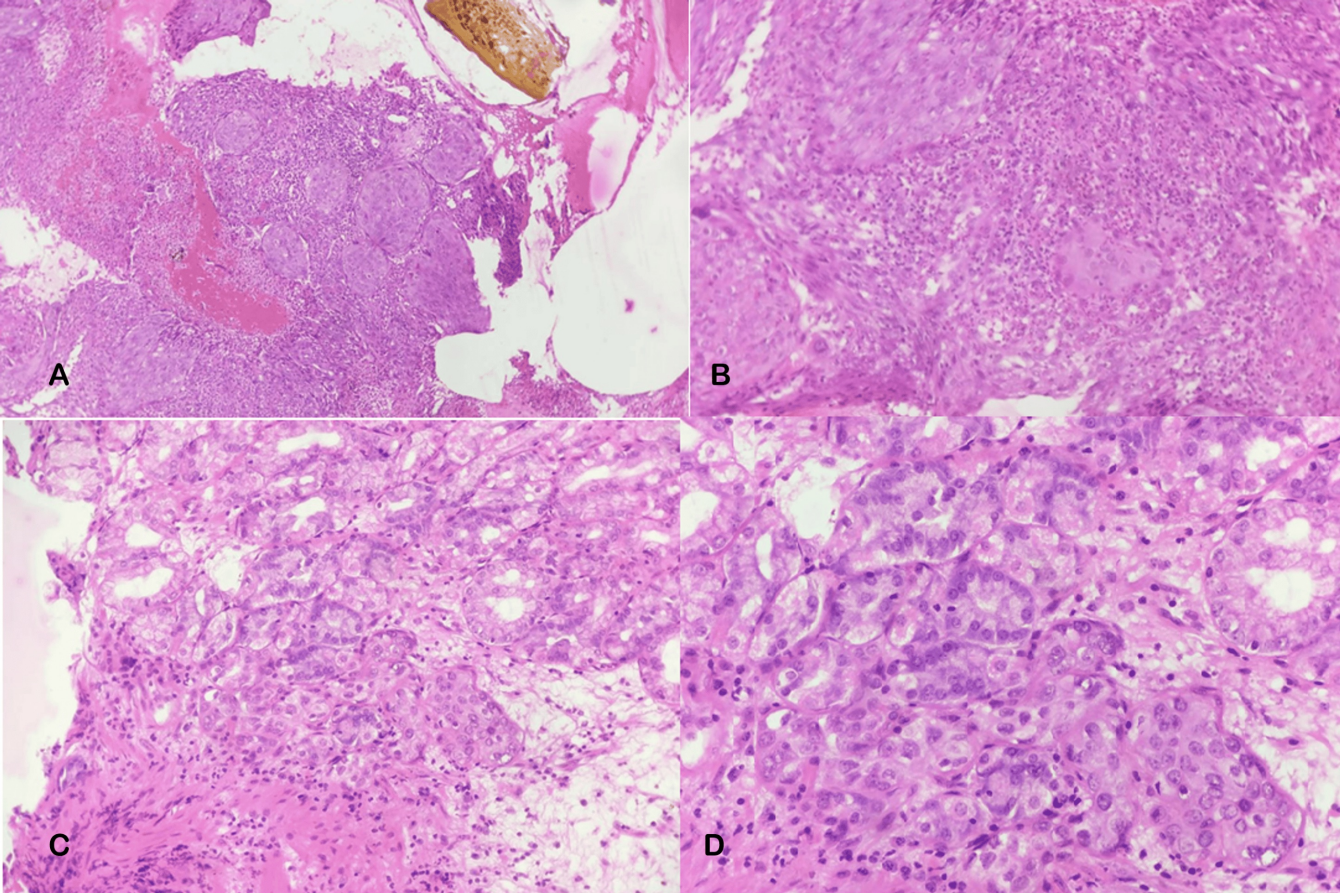
Histopathological images of gastric mucosal biopsies taken from the ulcerated lesion in the stomach. Histopathological images showing hematoxylin and eosin staining of gastric mucosal biopsy showing islands of malignant squamous cells in x200 (upper panel, A and B) and x400 (lower panel, C and D).

**Figure 3:**
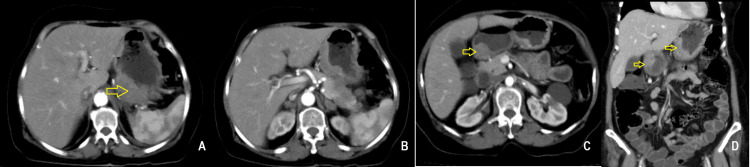
Contrast-enhanced computed tomography images in axial sections showing asymmetrical, heterogeneously enhancing wall thickening involving the proximal stomach along the lesser curvature (A), with focal infiltration of the pancreas (B), asymmetric thickening in the pylorus and duodenal bulb (C), and a coronal section showing involvement of both the lesser curvature and pyloroduodenum (D).

Esophagogastroduodenoscopy revealed an irregular friable ulcero-nodular lesion in the proximal body along the lesser curvature of the stomach, extending up to the angularis, with another irregular ulcerated malignant-appearing lesion in the pylorus and duodenum causing narrowing, across which the scope could not be negotiated (Figure 1). Significant food residue was noted due to pyloric obstruction. Biopsies were taken from the margin of the ulcer for histopathological examination, which showed ulcerated gastric mucosa overlying sheets of malignant cells with pleomorphic hyperchromatic nuclei, prominent nucleoli, moderate cytoplasm, an increased nuclear-to-cytoplasmic ratio, brisk mitosis, intratumoral neutrophils, focal spindling, and surrounding desmoplastic stroma with inflammatory cell infiltrate (Figure 2). P63 and CK7 were positive on immunohistochemistry. The biopsy was confirmative for non-keratinizing squamous cell carcinoma. Contrast-enhanced computed tomography of the abdomen revealed asymmetrical heterogeneously enhancing wall thickening with loss of mural stratification involving the gastroesophageal junction, cardia, and body along the lesser curvature, with focal infiltration of the pancreas, and another area of asymmetrical thickening in the pylorus and duodenal bulb (Figure 3). A few perigastric and peripancreatic lymph nodes were prominent adjacent to the antrum. After evaluation, she was diagnosed with gastric metastasis from primary cervical cancer, causing gastric outlet obstruction with chemotherapy failure.

During follow-up, as she was not tolerating oral feeds, a nasojejunal tube was placed for feeding. In view of her poor functional status and nutritional status, she was provided best supportive care. The patient succumbed to the illness after two months of palliative support.

## Discussion

Cervical cancer is one of the most common gynecological malignancies globally, with developing countries like India contributing one-fifth of annual new cases. The most common sites for metastases are the lungs, bone, liver, and brain, and the presentation is nonspecific [2]. The above case represents an unusual site of metastasis.

In our patient, a gastric lesion was detected on esophagogastroduodenoscopy (EGD), which appeared suspicious for malignancy, and histopathology confirmed squamous cell carcinoma. The diagnosis of primary gastric squamous cell carcinoma was excluded based on Parks criteria [3]. To meet these diagnostic criteria, three features are required: (a) the tumor should not be located at the cardia, (b) the tumor should not extend into the esophagus, and (c) the patient should not have evidence of squamous cell carcinoma (SCC) in any other part of the body. As the patient had a known source of SCC in the form of cervical cancer, primary gastric SCC was ruled out [3].

Metastasis of cervical cancer to the gastrointestinal tract (GIT) is rare, occurring in less than 4% of cases, with most reported cases discovered postmortem. A literature review of case reports of gastric metastasis published in 2022, which included 186 patients, showed breast cancer (29%) as the most common primary origin, followed by lung cancer (17.7%), renal cancer (10.7%), and malignant melanoma (10.2%). The average age was 59.1 years, with an age range of 56-71 years [1,4]. The metastases usually occurred in the middle or upper one-third of the stomach; one autopsy series reported that solitary lesions were more common than multiple [5].

Cervical cancer spreads by direct extension, transcoelomic, hematogenous, and, most commonly, lymphatic routes. As it does not have a direct route of spread to the gastrointestinal tract, cervical cancer metastasis to the stomach is rare. The most common site for GIT involvement in cervical cancer is the sigmoid colon by direct extension; the route of spread in the present case is unknown [6]. This patient had transmural involvement of the stomach wall with infiltration of the pancreas and ulceration of the proximal body and antropyloric mucosa, potentially indicative of a transcoelomic mode of spread, with initial involvement of the serosa and subsequent invasion of the remaining wall. Perigastric lymph nodes larger than 1 cm were identified before finding gastric metastases, which could indicate lymphatic involvement in cancer spread, either as the route for the spread or as a consequence of it [7].

Presentation of gastric metastases usually includes a combination of symptoms such as epigastric pain, vomiting, dysphagia, and fatigue. Gross blood loss in the form of hematemesis or melena, as seen in this case, is less common. Diagnosis in such cases is made by esophagogastroduodenoscopy to evaluate for upper gastrointestinal pathology. Biopsy and histopathological examination of the lesion, along with immunohistochemistry, play a critical role in identifying a lesion of metastatic origin. Subsequent imaging is done to look for other areas of metastasis and plan for treatment. Metastases to the stomach are rare and typically occur in the advanced stages of disease, with primary lesions spreading through the hematogenous route, in contrast to cervical cancer, which predominantly spreads through the lymphatic route [7].

To the best of our knowledge, only 11 prior cases of cervical cancer metastasizing to the stomach have been reported at the time of writing this report [8-18], as shown in Table 1.

**Table 1:** Case reports depicting the symptoms, location of gastric metastasis, outcome, stage of cervical cancer, and therapy offered.

First author	Age	Presentation	Location	Outcome	Stage of cervical cancer at diagnosis	Treatment completed at the time of detection of gastric metastasis	Treatment for gastric metastasis
Dhanushkodi et al. (8)	38	Epigastric discomfort	Antrum, corpus (lesser curvature)	N/A	Stage IIIB	Chemoradiotherapy + brachytherapy	Palliative chemotherapy and salvage gastrectomy
Moldovan et al (9)	49	Epigastric pain, vomiting, weight loss	Antrum	Disease-free at 9 months	Stage IIB	Surgery + chemoradioatherapy	Subtotal gastrectomy, Roux-en-Y, atypical hepatectomy, adjuvant chemoradiation
De Cicco et al (10)	81	UGI bleeding	N/A	Disease-free after 39 months	?	?	Salvage gastrectomy
Singhal et al (11)	48	Vomiting, hematemesis, epigastric pain	Fundus and corpus	Death	N/A	Panhysterectomy	None
Simones et al (12)	43	Anorexia, fatigue, melena	Cardia	Death	N/A	Surgery + chemoradiation	None
Oriuchi et al. (13)	59	Anorexia	Corpus and antrum (submucosal)	Death after 2 months	Stage IV	Untreated (newly diagnosed)	Palliation
Vural Topus et al. (14)	66	Incidental	Cardia and proximal corpus	N/A	N/A	Surgery (ECC+LEEP) + chemoradiation + brachytherapy	N/A
Kim et al. (15)	61	Incidental	Antrum	On chemotherapy after 12 months	Stage IV	Untreated (newly diagnosed)	Panhysterectomy + subtotal gastrectomy + adjuvant chemotherapy
Maglica et al. (16)	56	Incidental	Corpus	Deteriorating Health	Stage I/II	Panhysterectomy	Subtotal gastrectomy
Hasan et al. (17)	45	Dysphagia, vomiting	Corpus	N/A	Stage IV	Palliative chemoradiotherapy	Hospice/palliation
Al Ayoubi et al. (18)	46	UGI bleeding	Corpus (greater curvature) and duodenum	Death	Stage IV	Hysterectomy and chemoradiation	None

Most prior cases of gastric metastases of cervical cancer showed gastric wall thickening or submucosal nodules without invasion of neighboring organs or the serosa on imaging, similar to other causes of gastric metastases and more in line with a lymphatic mode of spread. The above case involved the proximal stomach and pylorus and infiltration of neighboring organs (pancreas), which were seen in only two other cases [8,9].

Gastric metastases from cervical cancer, as in other primary tumors, are more common in cancers at a later stage. In the literature, gastric metastases were present at presentation or found less than a year after the initial presentation in patients with cervical cancer stage IIIB or IV [8,13,15]. Most patients had received surgical, systemic, or a combination of both treatment modalities for cervical cancer before the development of gastric metastasis. Treatment in most cases was centered around palliation regardless of the initial stage of cervical cancer at presentation. While two of the ten cases [9,10] in the literature had disease-free survival at 9 and 39 months, respectively, other cases were not as fortunate despite receiving a similar intensity of treatment. Supportive treatment was centered around palliative chemotherapy and surgery if the patients required frequent blood transfusions or developed gastric outlet obstruction. Some of the cases presented with symptoms of obstruction, with vomiting or upper GI bleeding, but the majority had non-specific presentations in the form of anorexia or fatigue [8-18]. Obstructive symptoms and UGI bleeding may also be seen in the duodenal site of metastases but are more difficult to view on endoscopy [19,20]. More distal GI locations of metastases present with less acute obstruction, as seen in cases involving direct invasion of the sigmoid colon [6].

Palliation is the primary modality of treatment in metastatic disease. Though curative resection can be considered in cases of isolated or solitary gastric metastases, which have comparatively longer survival than multiple lesions, survival time is less than a year with a median of three months. Overall survival is based on the type and stage of the primary rather than the characteristics of the metastasis [4]. In the above case, while the stomach lesions appeared to be solitary ulcero-nodular lesions on EGD, imaging showed invasion of neighboring organs and enlargement of lymph nodes. She also had a history of failing first- and second-line chemotherapy regimens for cervical cancer. In view of metastatic disease, poor prognosis, and functional status, supportive palliation was the treatment chosen for this patient.

## Conclusions

In conclusion, our case highlights an unusual progression of cervical cancer metastasizing to the stomach, causing gastric outlet obstruction and gastrointestinal bleeding. Squamous cell carcinoma of the cervix with gastric metastasis is very rare and is associated with poor morbidity and mortality. It should be kept in mind that such progression may occur despite the advent of effective chemotherapeutic agents in the current era.
